# Cold Atmospheric Plasma Attenuates Breast Cancer Cell Growth Through Regulation of Cell Microenvironment Effectors

**DOI:** 10.3389/fonc.2021.826865

**Published:** 2022-01-17

**Authors:** Christos A. Aggelopoulos, Anna-Maria Christodoulou, Myrsini Tachliabouri, Stauros Meropoulis, Maria-Elpida Christopoulou, Theodoros T. Karalis, Athanasios Chatzopoulos, Spyros S. Skandalis

**Affiliations:** ^1^ Laboratory of Cold Plasma and Advanced Techniques for Improving Environmental Systems, Institute of Chemical Engineering Sciences, Foundation for Research and Technology Hellas (FORTH/ICE-HT), Patras, Greece; ^2^ Biochemistry, Biochemical Analysis & Matrix Pathobiology Res. Group, Laboratory of Biochemistry, Department of Chemistry, University of Patras, Patras, Greece

**Keywords:** CAP, breast cancer, estrogen receptor, tumor microenvironment, ROS, extracellular matrix

## Abstract

Breast cancer exists in multiple subtypes some of which still lack a targeted and effective therapy. Cold atmospheric plasma (CAP) has been proposed as an emerging anti-cancer treatment modality. In this study, we investigated the effects of direct and indirect CAP treatment driven by the advantageous nanosecond pulsed discharge on breast cancer cells of different malignant phenotypes and estrogen receptor (ER) status, a major factor in the prognosis and therapeutic management of breast cancer. The main CAP reactive species in liquid (i.e. H_2_O_2_, ${\text{NO}}_{2}^{-}{\text{/NO}}_{3}^{-}$ ) and gas phase were determined as a function of plasma operational parameters (i.e. treatment time, pulse voltage and frequency), while pre-treatment with the ROS scavenger NAC revealed the impact of ROS in the treatment. CAP treatment induced intense phenotypic changes and apoptosis in both ER+ and ER- cells, which is associated with the mitochondrial pathway as evidenced by the increased Bax/Bcl-2 ratio and cleavage of PARP-1. Interestingly, CAP significantly reduced CD44 protein expression (a major cancer stem cell marker and matrix receptor), while differentially affected the expression of proteases and inflammatory mediators. Collectively, the findings of the present study suggest that CAP suppresses breast cancer cell growth and regulates several effectors of the tumor microenvironment and thus it could represent an efficient therapeutic approach for distinct breast cancer subtypes.

## Introduction

Breast cancer is a highly heterogeneous disease that may exist in multiple subtypes. Its classification is critical for proper patient management and follow-up (1). Estrogen receptor alpha (ERα) expression profile is the prominent molecular feature for the discrimination of breast cancers, which are classified as ER-positive (ER+) and ER-negative (ER-). Almost 70% of breast cancers are ER+ and can be targeted with endocrine therapies. Others are classified as HER2-positive (they express the HER2 receptor tyrosine kinase) against which anti-HER2 targeted therapies are applied, and triple-negative breast cancer (TNBC, due to the absence of ER, progesterone and HER2 receptors), which has the worst prognosis and lacks an effective targeted therapy (2–4). Further, breast cancers can be classified with regard to their extracellular matrix (ECM) expression pattern resulting in distinct ECM subtypes related with different clinical outcome (5). Current options of breast cancer treatment include surgery, chemotherapy and radiotherapy, which all present severe limitations as they are not selective and offer incomplete tumor ablation. Therefore, the development of new therapeutic approaches that would ablate incurable breast tumor subtypes is urgently needed.

A rapidly evolving technology showing a strong potential for biomedical applications is cold plasma operating at atmospheric pressure (Cold Atmospheric Plasma, CAP) (6). CAP is generated from electrical energy allowing the creation of high energy electrons and reactive oxygen and nitrogen species (ROS/RNS) such as ^1^O_2_, OH radicals, NO_x_, atomic O, O_3_ and H_2_O_2_ which play critical role in transferring the reactivity from the gas discharge plasma zone to solutions/media (7), subsequently inducing specific biochemical responses. CAP has been used in several branches of modern medicine, such as in wound healing and sterilization (8). Notably, CAP has shown promising anticancer activity over the last decade (9–11). The *in vitro* application of this technology in numerous tumor cell lines resulted in the significant inhibition of cell growth, while it also attenuated the growth of subcutaneous xenograft tumors and melanoma in mice (9, 12, 13).

To date, many studies have concluded that the prominent factors contributing to the lethal CAP-mediated effects on cancer cells both *in vitro* and *in vivo* are the ROS/RNS generated during the complex interaction between plasma and cancer cells (14, 15). Fine tuning of the cellular levels of reactive species is critical for numerous cellular processes, such as metabolism, survival and differentiation, since they can act as mediators of various signaling pathways (16). Dysregulated ROS and/or RNS generation may have pro-tumorigenic effects by damaging nucleic acid and promoting genetic instability through induction of DNA strand breaks, which may induce the malignant transformation of tumor cells. On the other hand, excessive rise of intracellular ROS/RNS species appear anti-tumorigenic as they may induce irreversible damage in DNA, mitochondria and other vital components/structures of tumor cells as well as the activation of apoptotic mechanisms (16–20). In addition, several studies suggest the selective cancer cell apoptosis by CAP. For example, ROS produced in cancer cells at much higher levels than in normal cells, tend cancer cells to be more vulnerable than normal cells to ROS generated by CAP (21, 22).

Different setups for CAP cancer treatment have been extensively investigated with the prevailing devices being the plasma jet (9, 23, 24) and the dielectric barrier discharge (DBD) (22, 25, 26). Along with the basic principles of each device, the functions in the two devices are also different; by employing the jet device, the sample is faced as a separate, independent part of the system, while in the case of a DBD device, the sample constitutes a part of the discharge. Therefore, in case of a gentle treatment at a small area of the sample plasma jet device may be more suitable, while for treating a large area of sample and in a more intense mode, DBD could be considered more suitable (9). In addition, the main CAP discharge type reported to date includes AC-driven reactors. However, considering that the effectiveness of the treatment is closely related to the produced plasma reactive species, a rapid, effective and high production of reactive species is of eminent importance. In this context, recent efforts performed in reactors driven by nanosecond pulses (NSP) revealed its advantageous performance with respect to other plasma systems in terms of energy efficiency and production of plasma active particles (27).

In general, there are two different possible ways to perform a CAP-based cancer treatment, either through direct treatment of tumor cells or by an indirect treatment of culture solutions used for cells growth. In the present study, three approaches of DBD-CAP driven by the advantageous nanosecond pulsed discharge have been applied in breast cancer cells of different morphological and molecular features; the non-metastatic MCF-7/ER+ cells and the metastatic MDA-MB-231/ER- and Hs578T/ER- cells. The three DBD-CAP approaches included the direct treatment of the cell cultures, the indirect treatment of cell cultures using CAP-stimulated medium and the direct treatment of the cell cultures followed by the immediate change of the CAP-stimulated medium by fresh.

Collectively, we provide evidence that DBD-CAP suppresses breast cancer cell growth and metastatic potential by triggering cell apoptosis and regulating specific effectors of the tumor cell microenvironment such as ROS, matrix receptors, proteases and inflammatory mediators. The observed differential effects of the applied DBD-CAP approaches on the mammary tumor cell models used in the present study suggest that CAP-based technology could advance the therapy of distinct breast cancer subtypes of different ER profile and specific molecular features.

## Materials and Methods

### CAP Device and Treatment Strategy

**Figure 1:**
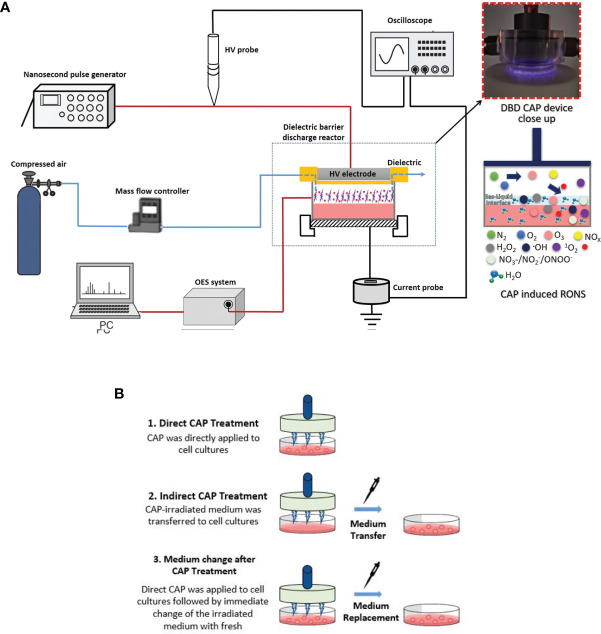
Schematic illustration of **(A)** Dielectric Barrier Discharge (DBD) CAP device; **(B)** breast cancer cell treatment approaches: 1. Direct DBD-CAP treatment (DBD-CAP was directly applied to cell cultures); 2. Indirect DBD-CAP treatment (DBD-CAP-treated medium was transferred to cell cultures); 3. Medium change after DBD-CAP treatment (Direct DBD-CAP was applied to cell cultures followed by immediate change of the CAP-treated medium by fresh untreated).

The depiction of the plasma device used to treat breast cancer cell lines along with a picture of the plane-to-plane DBD reactor operated with air is presented in **Figure 1A**. The HV electrode was a stainless-steel disc (28 mm diameter) covered by an acrylic glass cylinder (bottom thickness 1.5 mm, wall thickness 5 mm) acting as the discharge dielectric barrier. A petri dish (35 mm diameter) was filled with 1 mL of complete cell culture medium [Dulbecco’s Modified Eagle’s Medium (DMEM) with 10% FBS] and placed over a stainless-steel disc serving as the grounded electrode. The inter-electrode distance was ~6 mm, the distance between the dielectric surface and culture medium surface was ~1 mm and the air flow rate streamed above the culture medium surface was fixed at 1.0 liters per minute. The DBD reactor was driven by a high voltage (HV) generator (NPG-18/3500) able to produce positive nanosecond pulses (NSP) recorded on a Rigol MSO2302A, 300MHz oscilloscope using a Tektronix P6015A voltage probe and a Pearson electronics 2877, 300 Hz-200 MHz wideband current transformer. The average discharge power (*P*) was estimated by multiplying the pulse repetition rate (*f*) by the discharge pulse energy (*E_p_*), which was found by the integration of the instantaneous pulse voltage and current over the pulse duration (*t*) (28):


(1) $$P=f\;E_{p}=f\int_{pulse}V(t)I(t)dt$$

where V(*t*) and I(*t*) are the voltage and current signals, respectively.

Optical emission spectroscopy (OES) was employed to identify the main plasma-induced reactive oxygen and nitrogen species (ROS/RNS) in the gas phase. The emission spectra of the air-DBD was recorded with a fiber optics spectrometer (AvaSpec-ULS2048CL-EVO, Avantes). The NSP-DBD treatments were repeated at two different pulse voltages (27.6 and 31.2 kV), pulse repetition rates (0.5 and 1.5 kHz) and treatment times (2 to 6 min) at room temperature.

### Cell Culture and Reagents

Low metastatic MCF-7 (ER+) and highly metastatic MDA-MB-231 and Hs 578T (triple negative, ER-/PR-/HER2-) breast cancer cell lines (obtained from the ATCC) were utilized in the present study. For cell cultures, complete media (DMEM, #LM-D1109/500, Biosera) containing 10% fetal bovine serum and antimicrobial agents cocktail were used as described before (29). All chemicals were of the best commercially available grade.

### DBD-CAP Treatment Approaches on Breast Cancer Cells

The schematic illustration of the DBD-CAP treatment approaches applied in the present study is depicted in **Figure 1B**. In each experiment, a certain number of breast cancer cells (280,000 for MCF-7, 220,000 for MDA-MB-231 and Hs578T) was seeded on a 35-mm dish (#G203-35, Kisker) in complete medium (1 mL). After 24 h incubation under the standard cell culture conditions (a humidified, 37°C, 5% CO_2_ environment), the medium was replaced with 1 mL of fresh complete medium and the cell cultures were proceeded to DBD-CAP treatment. Three different treatment approaches were followed: (i) *Direct treatment*, where DBD-CAP was directly applied to cell cultures, (ii) *indirect treatment*, where cell-free DBD-CAP-treated medium (1 mL) was transferred to cell cultures, and (iii) *medium change*, where DBD-CAP was applied to cell cultures followed by immediate change of the CAP-treated medium by 1 mL of fresh untreated one. Following 24 h culture, cancer cells were proceeded to the various biochemical assays. For each treatment approach, a control group corresponding to cancer cells grown in 1 mL complete medium without DBD-CAP treatment was included.

### Preparation of N-Acetyl-L-Cysteine (NAC)-DMEM and Pre-Treatment of Breast Cancer Cells

DMEM containing NAC (NAC-DMEM) at concentration of 5 mM was made by dissolving NAC powder (Sigma-Aldrich, A7250) in complete medium (DMEM with 10% FBS). Breast cancer cells were cultured for 24 h, as described above, and the medium was replaced with 1 mL of NAC-DMEM. After 2 h, cell cultures were proceeded to the DBD-CAP treatments.

### pH and Temperature Measurement in Culture Media

Cell-free complete culture media were exposed to air NSP-DBD plasma for 2, 3, 4 and 6 min under the various abovementioned pulse voltages and pulse frequencies. Immediately after plasma treatment, the pH and temperature of the culture media were measured with a pH meter (Consort C830) and an infrared thermometer (Benetech GM900), respectively.

### Measurement of Reactive Species in Culture Media (H_2_O_2_, ${\text{NO}}_{2}^{-}$ and ${\text{NO}}_{3}^{-}$ ) and in Reactor Exhaust Gas (NO_x_)

The concentrations of H_2_O_2_, nitrate ions ${\text{(NO}}_{3}^{-})$ and nitrite ions ${\text{(NO}}_{2}^{-})$ in cell-free complete culture media at the various investigated DBD-CAP conditions were measured with the suitable QUANTOFIX^®^ test strips and quantified accurately with the QUANTOFIX^®^ Relax unit (Macherey-Nagel, GmbH). Moreover, NO_x_ concentration in the plasma exhaust gas was measured with a gas analyzer (Optima 7).

### Morphology/Phase Contrast Microscopy

For the observation of cell morphological changes, photographs of breast cancer cells (both the control groups and those subjected to the three DBD-CAP approaches) were captured at 3 h and 24 h after CAP treatment utilizing a color digital camera (CMOS) mounted on a phase contrast microscope (OLYMPUS CKX41, QImaging Micro Publisher 3.3RTV) through a 10x objective.

### Cell Viability

To evaluate cell viability, control and DBD-CAP-treated cells were stained with crystal violet solution followed by optical density measurement at 570 nm as described before (29). As an alternative method, breast cancer cells (control and DBD-CAP-treated cells cultured for 24 h) were detached from the 35-mm dishes with trypsin-EDTA 1× in PBS for 3 min, collected and counted on a hemocytometer counting chamber according to standard protocols.

### Fluorescence Microscopy

The assay was performed as described before (30). A green fluorescent phalloidin conjugate (Phalloidin-iFluor™ 488) (1:40, #00042, Biotium CF™488A) in 1% BSA/PBST was used to visualize actin cytoskeleton utilizing a fluorescent phase contrast microscope (OLYMPUS CKX41, QImaging Micro Publisher 3.3RTV) at 60×.

### RNA Isolation, cDNA Synthesis, and Real Time RT-PCR

RNA isolation from control and DBD-CAP-treated cells, cDNA synthesis and real time PCR analysis were conducted according to the manufacturer’s instructions. The relative mRNA expression of different gene transcripts (the corresponding primer sequences are presented in **Table 1**) was calculated as described in (30). The threshold cycle (Ct) number of each gene was normalized to the Ct of the normalizer (18S rRNA).

**Table 1:** Primer sequences used for quantitative RT-PCR.

Gene	Primer Sequence (5’-3’)	T_annealing_
*Bax*	Sense: CTGAGCGAGTGTCTCAAGCG	60°C
	Anti-Sense: CCCCAGTTGAAGTTGCCGTC	
*Bcl-2*	Sense: AAGAGCAGACGGATGGAAAAAGG	60°C
	Anti-Sense: GGGCAAATGCAAGTGAATG	
*CD44 total*	Sense: ATAATTGCCGCTTTGCAGGTGTATT	60°C
	Anti-Sense: ATAATGGCAAGGTGCTATTGAAAGCCT	
*uPA*	Sense: ACTACTACGGCTCTGAAGTCACCA	60°C
	Anti-Sense: GAAGTGTGAGACTCTCGTGTAGAC	
*MMP-1*	Sense: CCTCGCTGGGAGCAAACA	60°C
	Anti-Sense: TTGGCAAATCTGGCGTGTAA	
*MT1-MMP*	Sense: CATGGGCAGCGATGAAGTCT	60°C
	Anti-Sense: CCAGTATTTGTTCCCCTTGTAGAAGTA	
*IL-6*	Sense: TCCAGAACAGATTTGAGAGTAGTG	58°C
	Anti-Sense: GCATTTGTGGTTGGGTCAGG	
*IL-8*	Sense: GGCACAAACTTTCAGAGACAGCAG	61°C
	Anti-Sense: GTTTCTTCCTGGCTCTTGTCCTAG	
*18S RNA*	Sense: CAGGTCTGTGATGCCCTTAGA	60°C
	Anti-Sense: GCTTATGACCGGCACTTACTG	

### Western Blot Analysis

Western blot analysis was performed as described before (29). The primary antibodies used in the present study were: anti-PARP-1 (rabbit, 1:500, #ab6079, Abcam) and anti-α-tubulin (mouse, clone DM1A, 1:500, #T9026, Sigma-Aldrich). Detection of the proteins was performed according to the manufacturer’s instructions.

### Statistical Analysis

Reported values are expressed as mean ± standard deviation and are based on three (at least) independent experiments. GraphPad Prism unpaired t-test (95% confidence intervals) was used for the evaluation of statistical significant differences. Three significance levels are indicated, i.e. *p<0.05, **p<0.01 and ***p<0.001.

## Results

### Electrical and Optical Characteristics of the Discharge

**Figure 2:**
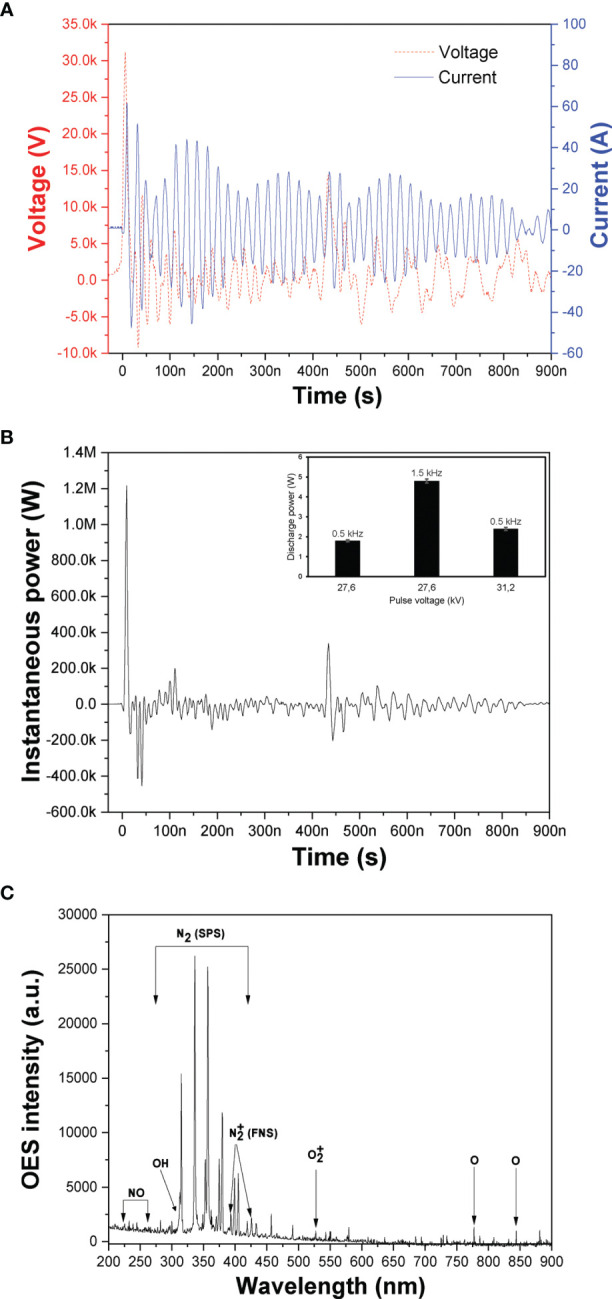
DBD-CAP characterization. **(A)** Instantaneous voltage and current NSP-DBD signals obtained at V=31.2 kV and *f* = 0.5 kHz; **(B)** Instantaneous power signal calculated by multiplying the instantaneous pulse voltage and current (inset: average discharge power as a function of pulse voltage and frequency; **(C)** Optical emission spectrum to identify the major excited ROS/RNS in the NSP-DBD with air gas.

The recorded NSP-DBD voltage and current waveforms during cell cultures treatment are shown in **Figure 2A**. Both voltage and current signals comprised by a sequence of pulses (31) with the rise time of the main positive HV pulse being ~4 ns and its duration ~15 ns at the full width at half maximum. The peak current was ~50 A resulted in an extremely high instantaneous power of ~1.2 MW **(** **Figure 2B** **)**, but at the same time the average discharge power was very low and ranged from 1.8 to 4.8 W under the investigated pulse voltages and frequencies (inset of **Figure 2B**) due to the quite low duty cycle involved.

Various excited molecular and atomic plasma ROS/RNS can be detected based on their peaks on the optical emission spectra (OES) of the discharge. The recorded spectrum at 31.2 kV is presented in **Figure 2C** revealing the major plasma species generated in the air NSP-DBD system of this study. Mainly, emission peaks originating from the N_2_ second positive system (N_2_ SPS) in the range 315-405 nm, the N_2_ first positive system (N_2_ FPS) between 500 and 900 nm, and ${\text{N}}_{2}^{+}$ at 393 and 427 nm were observed (27). Furthermore, OH radical emissions (309 nm) (32) which is a strong oxidant and precursor for H_2_O_2_ production, NO gamma emission lines in the range 230-260 nm (33), atomic oxygen (O) line at 777 and 844 nm, and ${\text{O}}_{2}^{+}$ line at 527 nm were detected (34).

### Quantification of Plasma-Generated NO_x_ in Gas Phase

During CAP process under air atmosphere, the dissolution of the produced gaseous NO_x_ in the cell culture media results in the formation of nitrites ${\text{(NO}}_{2}^{-})$ and nitrates ${\text{(NO}}_{3}^{-})$ that have been identified as reactive species playing a significant role in cancer cell death (35, 36). Thus, the concentration of the gaseous NO_x_ in the plasma exhaust gases under the different pulse voltages and frequencies were measured and presented in **Table 2**. NO concentration was not detected since in air-CAP nitrogen monoxide is rapidly converted to nitrogen dioxide (37) and therefore NO_x_ concentration is equal to NO_2_ concentration at each experimental condition. It is apparent that NO_2_ concentration increased with pulse voltage and repetition rate. At constant repetition rate of 0.5 kHz, NO_2_ concentration increased from 132 to 173 ppm when pulse voltage increased from 27.6 to 31.2 kV whereas at pulse voltage 27.6 kV and pulse frequency 1.5 kHz, NO_2_ concentration increased further to 239 ppm. The enhancement of the CAP-induced gaseous NO_2_ concentration with pulse voltage and frequency is directly related to the enhanced electric field and discharge power (inset of **Figure 2B**).

**Table 2:** Concentration of plasma-generated NO_x_ in the exhaust gases.

Conditions	NO (ppm)	NO_2_ (ppm)
0.5 kHz - 27.6 kV	–	132
0.5 kHz - 31.2 kV	–	173
1.5 kHz - 27.6 kV	–	239

### Quantification of Hydrogen Peroxide (H_2_O_2_), Nitrite ${\text{(NO}}_{2}^{-})$ and Nitrate ${\text{(NO}}_{3}^{-})$ in Cell-Free Culture Media

**Figure 3:**
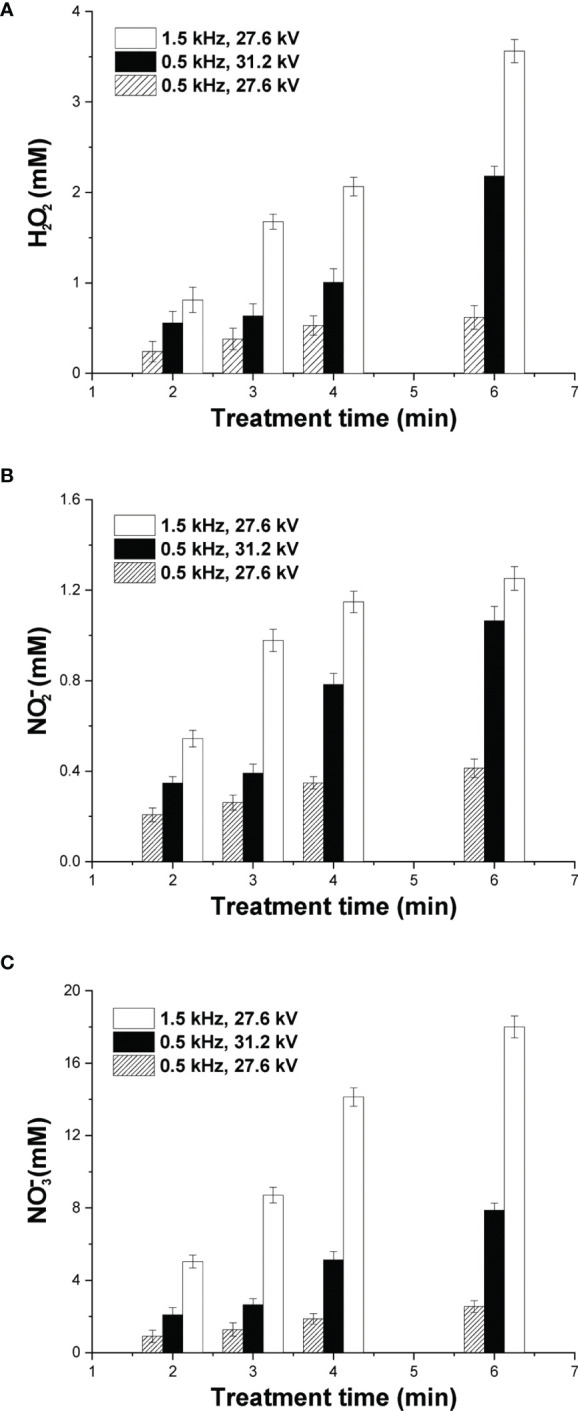
Quantification of major DBD-CAP-originated ROS/RNS. Concentration of **(A)** H_2_O_2_; **(B)** ${\text{NO}}_{2}^{-}$ ; **(C)** ${\text{NO}}_{3}^{-}$ in cell-free culture media (DMEM with 10% FBS) exposed to various plasma treatment times at different pulse voltages and frequencies. The values represent the mean ± SD of 3 (at least) independent experiments.

To investigate plasma-induced ROS/RNS generation in cell-free culture media, a quantitative analysis with emphasis on the species H_2_O_2_, ${\text{NO}}_{2}^{\neg }$ and ${\text{NO}}_{3}^{-}$ was performed. Besides nitrite and nitrate ions, hydrogen peroxide has been also identified as a major specie of cancer treatment (38). The concentration of H_2_O_2_ at various NSP-DBD treatment times and over two different values of the applied pulse voltage and pulse repetition rate is depicted in **Figure 3A**. H_2_O_2_ concentration increased significantly (i) with treatment time under all investigated conditions and (ii) with both pulse voltage and frequency. At mild treatment conditions (pulse voltage 27.6 kV and pulse frequency 0.5 kHz), the NSP-DBD for 2 min induced H_2_O_2_ generation of 0.24 mM which enhanced to 0.53 mM and 0.62 mM after 4 min and 6 min of treatment, respectively. Under strong treatment conditions (either pulse frequency 0.5 kHz/pulse voltage 31.2 kV or pulse frequency 1.5 kHz/pulse voltage 27.6 kV) after 6 min of plasma exposure time, H_2_O_2_ concentration in culture media increased from 0.62 mM to 2.18 mM (0.5 kHz/31.2 kV) and to 3.56 mM (1.5 kHz/27.6 kV).

The dissolution of the gaseous NO_2_ **(** **Table 2** **)** in the culture media results in the formation of nitrites, nitrates and hydrogen ions (39): 2 NO_2_ *(g)* + H_2_O → ${\text{NO}}_{2}^{-}$ *(aq)* + ${\text{NO}}_{3}^{-}$ *(aq)* + 2 H^+^ *(aq)*. The concentration of ${\text{NO}}_{2}^{-}{\text{ and NO}}_{3}^{-}$ in cell-free culture media at various plasma exposure times is shown in **Figures 3B, C**, respectively. Similar to H_2_O_2_, both RNSs increased substantially with treatment time, pulse voltage and pulse frequency, whereas ${\text{NO}}_{3}^{-}$ concentration was about one order of magnitude higher compared to that of ${\text{NO}}_{2}^{-}$ under all investigated plasma conditions. More specifically, at mild CAP conditions, the concentration of ${\text{NO}}_{2}^{-}$ was 0.21 mM after 2 min of treatment and increased to 0.41 mM at 6 min of treatment **(** **Figure 3B** **)**. Under strong DBD-CAP, ${\text{NO}}_{2}^{-}$ level was further increased to 1.07 mM at 6 min of treatment (0.5 kHz/31.2 kV) whereas an even higher ${\text{NO}}_{2}^{-}$ concentration was measured (1.25 mM) at higher pulse repetition rate (1.5 kHz/27.6 kV) for the same treatment time. ${\text{NO}}_{3}^{-}$ concentration exhibited an analogous increase with treatment time, pulse voltage and frequency with its maximum (18 mM) measured under strong DBD-CAP (1.5 kHz/27.6 kV) after 6 min **(** **Figure 3C** **)**.

### Estimation of pH and Temperature

**Figure 4:**
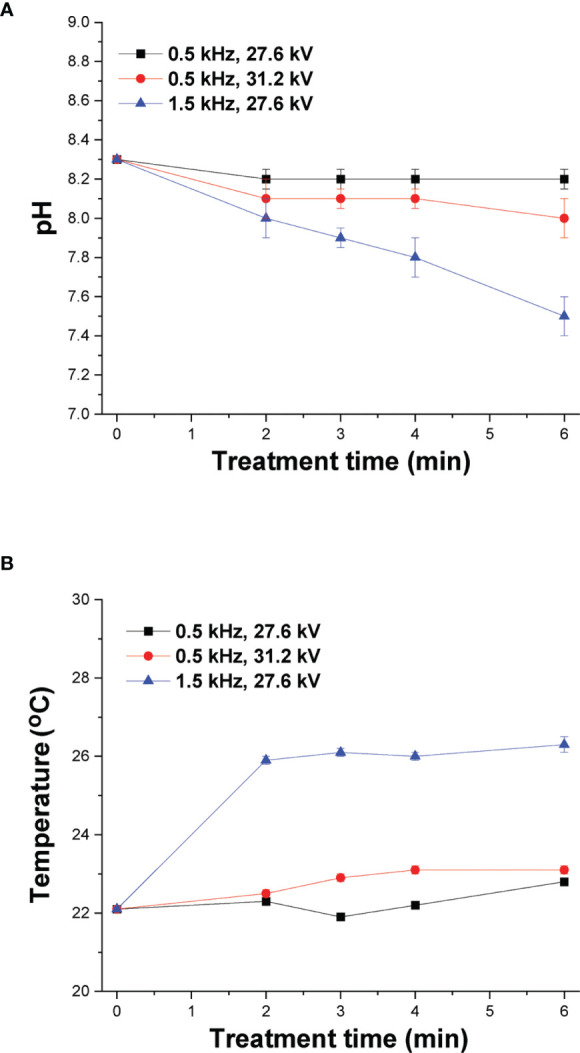
DBD-CAP treatment does not affect cell cultured medium pH and temperature. Effect of plasma exposure time on **(A)** pH and **(B)** temperature of cell-free culture media (DMEM with 10% FBS) at different pulse voltages and frequencies of the NSP-DBD device. The values represent the mean ± SD of 3 (at least) independent experiments.

It has been reported that the transfer of ROS/RNS generated by CAP in the aqueous phase affects the pH of the culture media and therefore it was measured for various treatment times under both mild and strong DBD-CAP **(** **Figure 4A** **)**. The pH of the cell-free culture media at 0.5 kHz was slightly changed from 8.3 (control) to 8.1 and 8.0 after 6 min of plasma treatment under both mild and strong DBD-CAP (pulse voltage 27.6 kV and 31.2 kV, respectively). At higher pulse frequency of 1.5 kHz (strong conditions), higher acidification was observed since pH was further decreased to 7.8 and 7.5 after 4 min and 6 min of treatment, respectively, which is due to the higher ROS/RNS concentration at 1.5 kHz compared to that at 0.5 kHz as already shown in **Figure 3**.

Another important parameter during cancer cells treatment by CAP is the change in temperature of culture media since temperature increase can inflict thermal damages in cells (40). The temperature of cell culture media was measured at various treatment times (2, 3, 4 and 6 min) of CAP treatment and 0.7 ± 0.1°C increase was detected after 6 min under mild DBD-CAP, whereas the corresponding increase under strong DBD-CAP (0.5 kHz/31.2 kV) was 1.0 ± 0.1°C **(** **Figure 4B** **)**. Therefore, the NSP-DBD device of this study operating even under strong treatment conditions overcomes the heating effect of other AC-driven plasma devices (41). Nevertheless, a higher increase of 4.2 ± 0.2°C was observed at 1.5 kHz/27.6 kV **(** **Figure 4B** **)**.

### DBD-CAP Attenuates Breast Cancer Cell Growth

**Figure 5:**
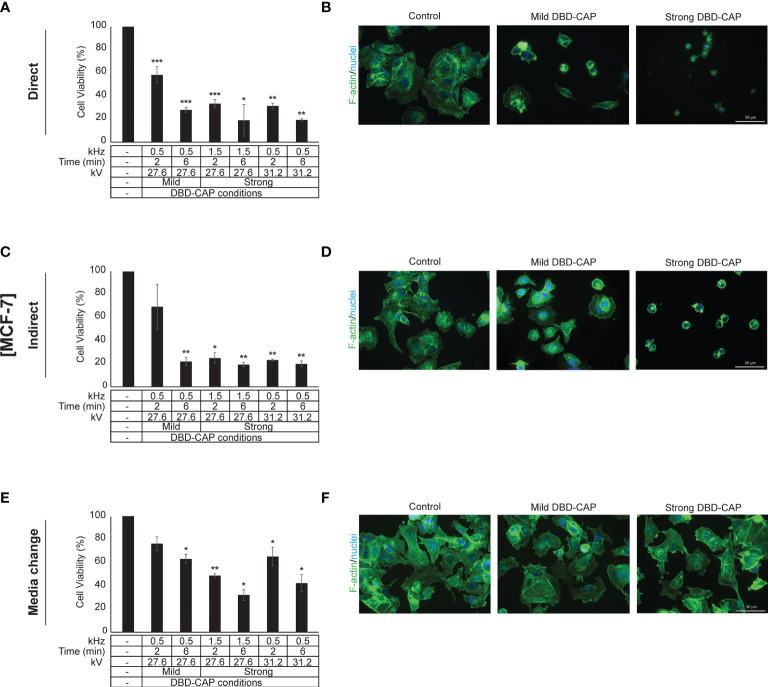
DBD-CAP suppresses MCF-7 (ER+, low metastatic) breast cancer cell growth. **(A**, **C**, **E)** Assessment of % cell survival 24 h after treatment with mild (0.5 kHz/27.6 kV) or strong (1.5 kHz/27.6 kV or 0.5 kHz/31.2 kV) DBD-CAP (direct, indirect, media change) for 2 and 6 min. The values represent the mean ± SD of 3 (at least) independent experiments. Asterisks illustrate significant differences of the different treatment conditions compared to untreated (control) cells (*p < 0.05, **p < 0.01, ***p < 0.001). **(B, D, F)** Cell morphology after mild (0.5 kHz/27.6 kV) or strong (0.5 kHz/31.2 kV) DBD-CAP treatment for 2 min. Scale bars, 50 µm. Representative immunofluorescence images are shown (*F-actin*, green; *nuclei*, blue/DAPI).

**Figure 6:**
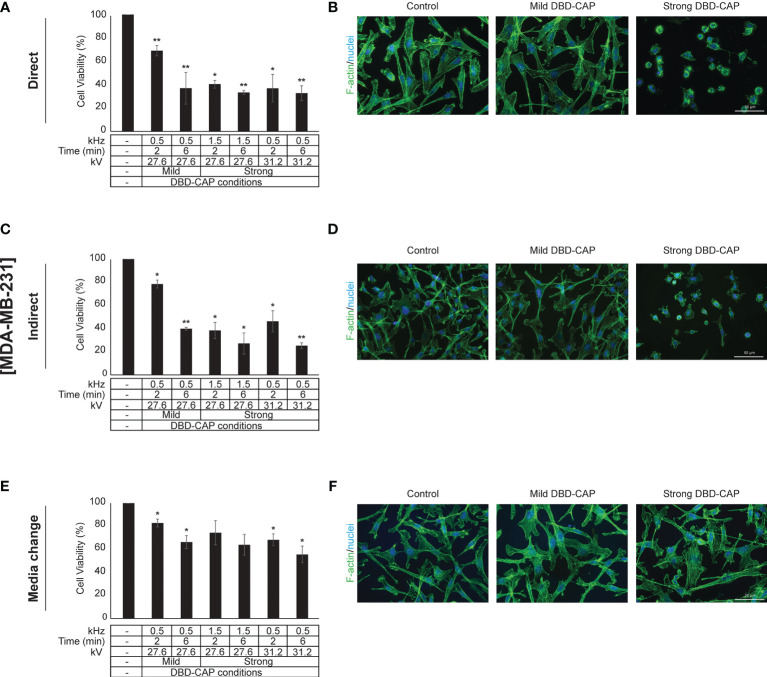
DBD-CAP suppresses MDA-MB-231 (ER-, highly metastatic) breast cancer cell growth. **(A, C, E)** Assessment of % cell survival 24 h after treatment with mild (0.5 kHz/27.6 kV) or strong (1.5 kHz/27.6 kV or 0.5 kHz/31.2 kV) DBD-CAP (direct, indirect, media change) for 2 and 6 min. The values represent the mean ± SD of 3 (at least) independent experiments. Asterisks illustrate significant differences of the different treatment conditions compared to untreated (control) cells (*p < 0.05, **p < 0.01). **(B, D, F)** Cell morphology after mild (0.5 kHz/27.6 kV) or strong (0.5 kHz/31.2 kV) DBD-CAP treatment for 2 min. Scale bars, 50 µm. Representative immunofluorescence images are shown (*F-actin*, green; *nuclei*, blue/DAPI).

**Figure 7:**
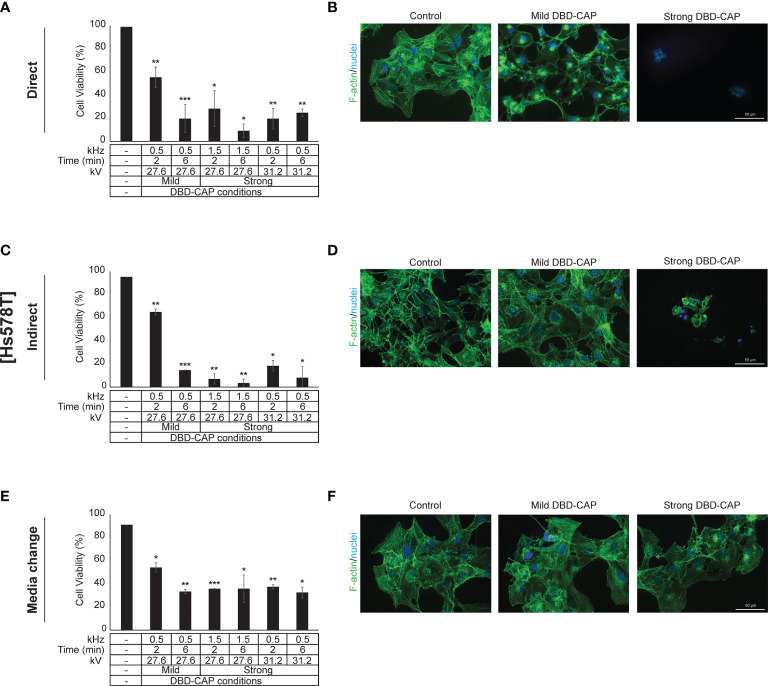
DBD-CAP suppresses Hs578T (ER-, highly metastatic) breast cancer cell growth. **(A**, **C**, **E)** Assessment of % cell survival 24 h after treatment with mild (0.5 kHz/27.6 kV) or strong (1.5 kHz/27.6 kV or 0.5 kHz/31.2 kV) DBD-CAP (direct, indirect, media change) for 2 and 6 min. The values represent the mean ± SD of 3 (at least) independent experiments. Asterisks illustrate significant differences of the different treatment conditions compared to untreated (control) cells (*p < 0.05, **p < 0.01, ***p < 0.001). **(B**, **D**, **F)** Cell morphology after mild (0.5 kHz/27.6 kV) or strong (0.5 kHz/31.2 kV) DBD-CAP treatment for 2 min. Scale bars, 50 µm. Representative immunofluorescence images are shown (*F-actin*, green; *nuclei*, blue/DAPI).

Direct treatment of MCF-7 (ER+, low metastatic), MDA-MB-231 and Hs578T (ER-, highly metastatic) breast cancer cells with mild (0.5 kHz/27.6 kV) or strong (1.5 kHz/27.6 kV or 0.5 kHz/31.2 kV) DBD-CAP conditions revealed a time- and dose-dependent effect on cell viability. Specifically, short (2 min) mild DBD-CAP induced 30-40% toxicity to all cell lines, which was further increased up to 60-80% when cells were subjected to longer (6 min) mild DBD-CAP treatment. Strong DBD-CAP of short treatment time (2 min) resulted in 60-80% cytotoxicity (at levels similar to those observed for the long mild DBD-CAP), which was further enhanced up to 70-90% when cells were subjected to strong DBD-CAP for 6 min **(** **Figures 5A**, **6A**, **7A** **)**. Indirect treatment resulted in a similar strong effect on cell viability as shown by the increased cytotoxicity at all DBD-CAP conditions used in the present study **(** **Figures 5C**, **6C**, **7C** **)**. Among cancer cells, Hs578T cells showed the highest toxicity (approx. 90%) when subjected to strong indirect DBD-CAP even with short treatment time (2 min) **(** **Figure 7C** **)**. On the other hand, MDA-MB-231 cells showed a more resistant phenotype at both direct and indirect treatment approaches compared to the other two cell lines **(** **Figures 6A, C** **)**.

To further investigate these observations, breast cancer cells were directly subjected to DBD-CAP under the same conditions as in direct/indirect treatment approaches and the culture medium was immediately replaced with fresh. This approach (medium change) resulted in the lowest cytotoxicity at all three breast cancer cell lines **(** **Figures 5E**, **6E**, **7E** **)**. This was particularly evident for Hs578T cells, which exhibited remarkably lower cytotoxicity (approx. 60%) when medium was changed after strong DBD-CAP **(** **Figure 7E** **)** compared to direct or indirect approaches **(** **Figures 7A, C**, respectively) where cytotoxicity ranged from approx. 70 to 95%. On the other hand, the same approach revealed a significantly lower cytotoxicity for MDA-MB-231 cells **(** **Figure 6E** **)** which were the most resistant to all applied approaches.

Overall, these results revealed an important role of DBD-CAP-originated species within the medium in the inhibition of breast cancer cell growth.

### DBD-CAP Affects Breast Cancer Cell Morphology

The observed significant reduction in cell viability prompted us to examine cell cytoskeleton changes upon DBD-CAP treatment. To this aim, we used Phalloidin-Fluor488 to visualize filamentous actin (F-actin). Treatment of cells with direct or indirect mild DBD-CAP (2 min) resulted in a moderate to strong actin cytoskeleton disorganization, which was more evident in MCF-7 **(** **Figures 5B, D** **)** and Hs578T **(** **Figures 7B, D** **)** cells. The changes in filamentous actin organization were significantly intensified in all three breast cancer cell lines upon treatment with direct or indirect strong DBD-CAP (2 min), which was followed by cell rounding and a dramatic loss of cells as expected **(** **Figures 5**, **6**, **7B, D** **)**. On the other hand, only moderate changes in actin cytoskeleton were observed when the CAP-treated medium was immediately replaced by fresh (medium change approach) at both mild and strong DBD-CAP conditions while no significant loss of cells occurred in line with the low cytotoxicity observed for this approach **(** **Figures 5**, **6**, **7F** **)**.

### Pretreatment With NAC Partly Weakens DBD-CAP-Mediated Effects on Breast Cancer Cell Viability

**Figure 8:**
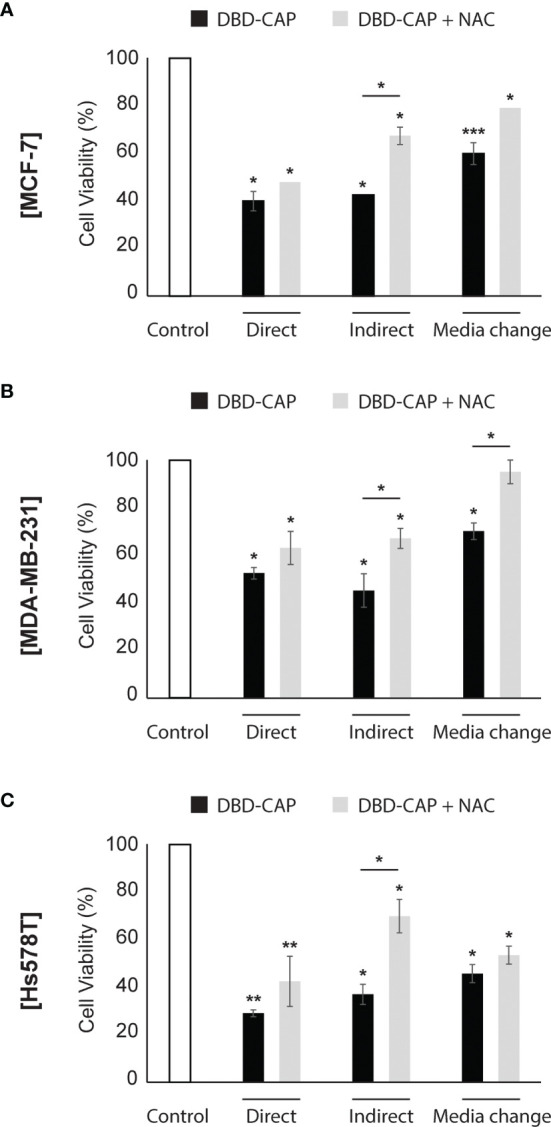
ROS scavenger NAC partly inhibits DBD-CAP-mediated effects on breast cancer cell viability. Assessment of % cell survival 24 h after treatment of **(A)** MCF-7, **(B)** MDA-MB-231, **(C)** Hs578T with mild (0.5 kHz/27.6 kV) DBD-CAP (direct, indirect, media change) for 4 min in the absence or presence of NAC (pre-treatment with 5 mM NAC-DMEM for 2 h). The values represent the mean ± SD of 3 (at least) independent experiments. Asterisks illustrate significant differences of the different treatment conditions compared to untreated (control) cells as well as between DBD-CAP and DBD-CAP + NAC (*p < 0.05, **p < 0.01, ***p < 0.001).

It is known that the CAP-originated reactive species are the major anti-cancer effectors inhibiting the growth of tumor cells *in vitro.* Therefore, to further investigate the strong anti-cancer effect of DBD-CAP treatment, breast cancer cells were treated with the intracellular ROS scavenger NAC (5 mM) prior to mild (0.5 kHz/27.6 kV) DBD-CAP (direct, indirect or media change) for 4 min. The results revealed that NAC partly counteracted the significant reduction in cell viability, especially when indirect treatment was applied to breast cancer cells **(** **Figure 8** **)**. These findings were confirmed by phase contrast microscopy, which revealed negligible changes in cell morphology when CAP-treated cells were pretreated with the ROS scavenger compared to the CAP-treated cells alone **(** **Supplementary Figure 1** **)**.

### DBD-CAP Induces the Mitochondrial Apoptotic Pathway in Breast Cancer Cells

**Figure 9:**
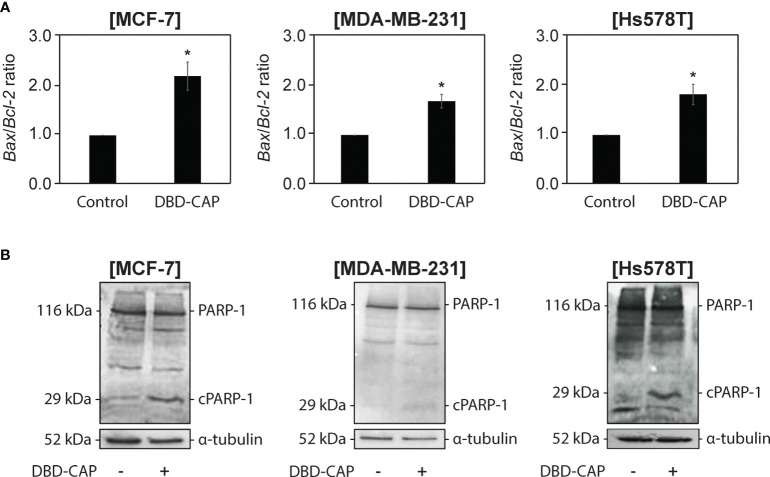
DBD-CAP induces apoptosis in both ER+ and ER- breast cancer cells. **(A)** *Bax/Bcl-2* ratio as estimated from the relative expression of *Bax* and *Bcl-2* to *18S rRNA* (housekeeping gene) mRNA levels by quantitative qPCR analysis in MCF-7/ER+, MDA-MB-231/ER- and Hs578T/ER- cells treated with mild (0.5 kHz/27.6 kV) direct DBD-CAP for 4 min. The values represent the mean ± SD of 3 (at least) independent experiments. Asterisks illustrate significant differences between control (untreated) and DBD-CAP-treated cells (*p < 0.05). **(B)** Immunoblot analyses of PARP-1/cPARP-1 and α-tubulin (loading control) in MCF-7/ER+, MDA-MB-231/ER- and Hs578T/ER- untreated (left lane) or treated with mild (0.5 kHz/27.6 kV) direct DBD-CAP for 4 min (right lane). Representative blots are shown.

To examine whether the significant inhibition of breast cancer cell growth after DBD-CAP treatment was due to induction of cell apoptosis, we examined the mRNA levels of two key apoptosis-associated mitochondrial proteins, namely the proapoptotic Bax and the antiapoptotic Bcl-2, and evaluated the changes in their ratio since an elevated Bax/Bcl-2 ratio indicates the induction of the intrinsic cell apoptotic pathway (42). The results showed that the Bax/Bcl-2 ratio was significantly elevated (1,5- to 2,5-fold) in all three cell lines subjected to mild (0.5 kHz/27.6 kV) direct DBD-CAP compared to untreated cells (control) **(** **Figure 9A** **)**. To further investigate this observation, we performed immunoblotting analysis for poly [ADP-ribose] polymerase 1 (PARP-1) protein (43). Notably, DBD-CAP induced PARP-1 cleavage, a marker of apoptosis, as evidenced by the detection of the PARP-1 fragment (29-kDa, cPARP-1) in all cell lines **(** **Figure 9B** **)**.

### DBD-CAP Affects the Expression of Matrix Effectors

**Figure 10:**
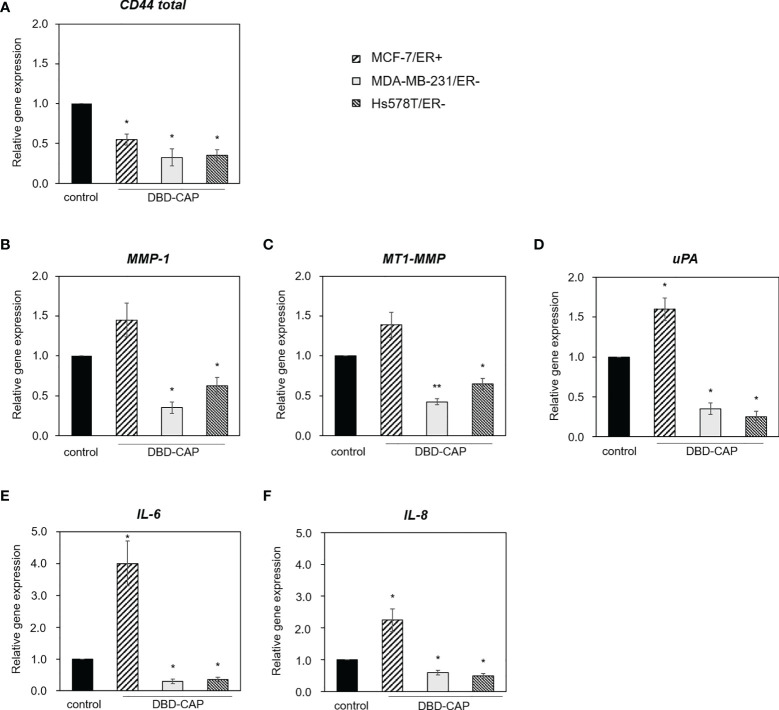
DBD-CAP regulates the expression of matrix effectors in breast cancer cells. Quantitative qPCR analysis of **(A)** *CD44*, **(B)** *MMP-1*, **(C)** *MT1-MMP*, **(D)** *uPA*, **(E)** *IL-6* and **(F)** *IL-8* in MCF-7/ER+, MDA-MB-231/ER- and Hs578T/ER- cells treated with mild (0.5 kHz/27.6 kV) direct DBD-CAP for 4 min. The values represent the mean ± SD of 3 (at least) independent experiments. Asterisks illustrate significant differences between control (untreated) and DBD-CAP-treated cells (*p < 0.05, **p < 0.01).

Next, we investigated whether DBD-CAP would affect tumor cell microenvironment. To this aim, we treated cells with mild (0.5 kHz/27.6 kV) direct DBD-CAP that caused a moderate cytotoxicity and examined the expression of key matrix molecules involved in breast tumor cell malignant properties by qPCR analysis. Our findings showed that DBD-CAP resulted in a significant down-regulation of CD44 (the main cellular receptor for the polysaccharide hyaluronan and a prominent cancer stem cell marker) mRNA expression in all three breast cancer cell lines **(** **Figure 10A** **)**. Further, DBD-CAP treatment significantly modulated the expression of several proteases including matrix metalloproteases (MMP-1 and the membrane-associated MT1-MMP) as well as uPA, a critical component of the plasminogen activation system that promotes MMP activation and degradation of most ECM proteins (**Figures 10B–D**, respectively). Notably, DBD-CAP treatment showed an opposite effect in the expression of the proteases between ER- and ER+ breast cancer cells. Specifically, DBD-CAP significantly down-regulated the mRNA levels of MMP-1, MT1-MMP and uPA in MDA-MB-231/ER- and Hs578T/ER- cells, while it up-regulated these proteases in MCF-7/ER+ cells. In a similar mode of action, DBD-CAP differentially affected the expression of inflammatory mediators such as interleukins IL-6 and IL-8 since they were also up-regulated in MCF-7/ER+ cells, while they were significantly suppressed in ER- cells (MDA-MB-231 and Hs578T) **(** **Figures 10E, F** **)**.

## Discussion

Breast cancer derives from a number of distinct tumors of epithelial cells of the breast and it cannot accurately descried as a single disease (44, 45). ERα expression is a major criterion for the classification and clinical management of breast cancers. Although a high proportion is ER+ and responsive to antiestrogens-based treatment, ER- breast cancers (such as TNBC) still lack effective targeted therapies while they present high risk of recurrence and the worst prognosis. Conventional treatments for controlling cancer is mostly based on anti-cancer agents, exhibiting limited effectiveness, high-cost and numerous side-effects. It is therefore obvious that there is urgent need to adopt new technologies and methods to deal with this problem. The outstanding performance of CAP concerning its anti-cancer effects has already been shown in a number of *in vitro* studies for the treatment of different types of tumors (46, 47), including those of skin (33, 40, 48), pancreatic (49), colon (50), lung (51, 52) and breast (53–55). Nevertheless, although these studies have indicated the prevailing character of plasma as a promising tool in tumor therapy, the responsible effectors and the underlying molecular mechanisms remain unclear.

The critical role of ER in breast cancer pathobiology prompted us to design this study with the assumption that ER would provide a differential outcome on breast cancer subtypes after treatment with plasma. Thus, we investigated the effects of DBD-CAP on three breast cancer cell models of distinct ER status (MCF-7/ER+, MDA-MB-231/ER- and Hs578T/ER-).

The chemical factors in CAP alleged as the critical anti-cancer effectors involve the reactive species generated during plasma process (14, 56). Many studies highlight that the CAP-treated medium, defined as a solution containing most of the long-lived reactive species deriving from CAP, are capable to cause strong and selective anti-cancer effect both *in vitro* and *in vivo* (33, 52, 57–62). Long-lived reactive oxygen and nitrogen species (ROS/RNS), including H_2_O_2_, ${\text{NO}}_{2}^{-}$ /HNO_2_ and ${\text{NO}}_{3}^{-}$ /HNO_3_, and O_3_ possessing long-lasting halftimes, are critical for the biochemical stimulation of plasma-activated medium (63). Peroxynitrite/peroxynitrous acid (ONOO^-^/ONOOH) in CAP-treated medium has been also recognized as a quite effective agent based on chain reactions producing NO which is a beneficial specie for pathogen inactivation/cell apoptosis (Eqs. 1-10).


(1) ·N+·OH→·NO+·H

(2) $$\cdot \text{H}+{\text{NO}}_{2}^{-}\to \cdot \text{NO}+{\text{OH}}^{-}$$

(3) $${\text{N}}_{2}{\text{O}}_{3}↔\cdot \text{NO}+\cdot {\text{NO}}_{2}$$

(4) $${\text{HNO}}_{2}+\cdot \text{H}\to \cdot \text{NO}+{\text{H}}_{2}\text{O}$$

(5) $${\text{HNO}}_{2}+{\text{H}}_{2}{\text{O}}_{2}\to \text{ONOOH}↔\cdot {\text{NO}}_{2}+\cdot \text{OH}$$

(6) $${\text{NO}}_{2}^{-}+{\text{H}}_{2}{\text{O}}_{2}\to {\text{ONOO}}^{-}↔\cdot \text{NO}+\cdot {\text{O}}_{2}^{-}$$

(7) $$\cdot \text{OH}+\text{ONOOH}\to \cdot \text{NO}+{\text{O}}_{2}+{\text{H}}_{2}\text{O}$$

(8) $$\cdot {\text{NO}}_{2}+.\text{O}\to \cdot \text{NO}+{\text{O}}_{2}$$

(9) $$\cdot \text{N}{\text{O}}_{2}+\cdot \text{N}\to 2\;\cdot \text{NO}$$

(10) $${\cdot \text{NO}}_{2}+{\text{O}}_{3}\to \cdot \text{NO}+{\text{O}}_{2}$$

The major ROS/RNS species generated in the gaseous plasma phase of our NSP-DBD system identified as N_2_(SPS), ${\text{N}}_{2}^{+}$ , OH, O, NO_2_/NO_x_ and ${\text{O}}_{2}^{+}$ **(** **Figure 2C** **)** resulted in the formation of strong oxidants inside the cell culture media (e.g. H_2_O_2_, ${\text{NO}}_{2}^{-}$ and ${\text{NO}}_{3}^{-}$ ). These species were found to increase substantially with treatment time, pulse voltage and pulse frequency **(** **Figure 3** **)** similarly to gaseous NO_2_ concentration **(** **Table 2** **)**, which is indicative of the increase of ROS/RNS concentration at higher discharge power (inset of **Figure 2B**).

Transferring plasma inside the body is an important and challenging issue especially for tumors located in deeper areas. The present study deals with this issue by evaluating the effectiveness of three different CAP approaches (direct, indirect and media change) and clearly shows that indirect treatment of the cells (exposure of cells with DBD-CAP-stimulated medium) induced similar or, in some cases, higher cytotoxicity than the direct approach **(** **Figures 5**, **6**, **7** **)**. Therefore, the indirect approach could be used to treat deep tumors (including breast cancer) by injecting plasma-activated medium alone or in combination with a conventional therapy highlighting the important perspective to apply this technology in clinical practice in a more simplified way without directly subject the patients to plasma treatment. To further understand in depth the CAP-mediated mechanisms it is also useful to examine the cytotoxic effect of CAP treatment by renewing the medium immediately after CAP treatment. In our study, the observation that the medium change approach caused significant lower cytotoxicity to all breast cancer cell lines further supports the assumption that the cytotoxicity of CAP depends on the CAP-derived ROS/RNS (59).

Over the past decade, the importance of the plasma ROS/RNS in cytotoxicity has been investigated (9, 62, 64, 65). To that end, the most effective way to reveal the significance of the reactive species is by using specific chemical species (known as scavengers) added in the medium which can eliminate the CAP effect. CAP-generated ROS/RNS are trapped by these scavengers showing indirectly their importance in CAP treatment (66). In line with this, in the present study, we demonstrate that DBD-CAP-generated ROS contribute to the observed cytotoxicity since pre-treatment of the tumor cells with the ROS scavenger NAC weakened the toxic effects of the plasma **(** **Figure 8**, **Supplementary Figure 1** **)**.

With regard to cell morphology, in agreement with previous studies (67, 68), shortly after the treatment with plasma (direct or indirect) many tumor cells underwent morphological changes that ranged from moderate to strong depending on the cell type and the applied DBD-CAP conditions. Their shapes were changed from spread to contractive accompanied by a disorganized actin cytoskeleton and loss of cell polarization and cytoplasmic protrusions **(** **Figures 5**, **6**, **7B, D** **)**. The observed changes were dependent on plasma-derived reactive species since both the immediate change of the DBD-CAP-stimulated medium (medium change approach) **(** **Figures 5**, **6**, **7F** **)** and the treatment of tumor cells with the ROS scavenger NAC prior to DBD-CAP treatment **(** **Supplementary Figure 1** **)** resulted in minor cell morphological changes. As a consequence, DBD-CAP might affect proteins that are located on tumor cell membranes, including receptors, channels and transporters, thus affecting cell-cell and cell-matrix interactions and related functions. However, this remains to be investigated.

Regarding the physical parameters, the final temperature and pH of the medium covering the tumor cells is determined by its initial temperature or pH, composition, CAP treatment dose and the power of discharge (9, 41). In our experimental set up, temperature changes were not high enough to impose thermal damages in cells **(** **Figure 4B** **)**. Since the standard temperature in the incubator is 37°C, the slightly warmed medium after CAP treatment does not affect tumor cell growth and viability. Another important parameter that should be considered in order to understand the chemical essence of DBD-CAP cytotoxicity is pH. A slight acidification was detected especially when cells were subjected to higher pulse frequency (1.5 kHz/27.6 kV) due to the higher RNS (HNO_2_/HNO_3_/ONOOH) concentration at these conditions **(** **Figure 4A** **)**. The negligible changes in the pH of the medium could be due to the existence of buffering chemicals in the media and are obviously not harmful for the cells in line with previous studies (9, 41).

CAP induces cancer cell death mainly *via* induction of apoptosis (11, 69). Apoptotic pathways may be extrinsic, which acts on cell surface death receptors, or intrinsic, which acts through mitochondria. The latter pathway is regulated by key apoptosis-related proteins, including cytochrome *c*, Bax and Bcl−2, which subsequently activate caspase−3 and PARP-1 protein (70). Bax translocates to the interior of mitochondria upon apoptosis induction and is important for apoptotic signaling (71). On the other hand, Bcl-2 protein negatively regulates cell apoptosis since it promotes cell survival (72). Notably, the ratio of Bax to Bcl-2 indicates induced or suppressed cell apoptosis rates (73). In this study, DBD-CAP resulted in the increase of the Bax/Bcl-2 ratio in both ER+ and ER- breast cancer cells **(** **Figure 9A** **)** suggesting that the observed cell apoptosis is dependent on alterations of these proteins expression and is associated with the mitochondrial apoptotic pathway. This is further supported by the finding that DBD-CAP induced degradation of PARP-1, which is also a marker of cell apoptosis **(** **Figure 9B** **)**. Therefore, it is likely that upon rise of the Bax/Bcl−2 ratio in the mitochondrial membrane, cytochrome *c* is translocated from the mitochondria to the cytosol, resulting in activation of specific proteases (such as caspase−3) that cleave PARP-1. Interestingly, inhibition of PARP-1 activity compromises base excision repair and promotes synthetic lethality in tumor cells with homologous recombination (HR) defects. However, this is not the case for normal cells since they retain the ability to repair DNA through HR (74). These observations are consistent with the possibility that DBD-CAP selectively ablates tumor cells and indicate the potential of CAP as a promising anti-cancer treatment modality.

The present study suggests that the tumor-suppressive properties of DBD-CAP on mammary carcinoma cells occur, at least in part, due to modulation of the tumor cell microenvironment. Our findings revealed significant changes in the expression of specific extracellular matrix effectors with established roles in tumor growth and metastasis. In particular, DBD-CAP markedly down-regulated CD44, which is the major cellular receptor of the extracellular heteropolysaccharide hyaluronan, in both ER+ and ER- breast cancer cells **(** **Figure 10A** **)**. Hyaluronan-CD44 interactions have a substantial impact on stemness properties of cancer stem cells and drug resistance through promotion of the epithelial-to-mesenchymal transition program, epigenetic control, oxidative stress resistance, and secretion of exosomes/extracellular vesicles (75). Given that CD44 is a prominent breast cancer stem cell marker (76), our results suggest that CAP affects stem cell niche and suppresses breast cancer cell stemness. DBD-CAP was shown to affect also the proteolytic potential of tumor cells since it significantly down-regulated the expression of MMP-1, MT1-MMP and uPA in the metastatic ER- breast cancer cells **(** **Figures 10B–D** **)**. These proteases have been shown to be involved in cancer dissemination. For example, membrane-associated MT1-MMP, a key effector in invadopodia-dependent ECM degradation, interacts with CD44 in protrusions/invadopodia of ER- cells thus promoting proteolytic activities and tumor cell metastasis (77). Moreover, the inhibitory effect of DBD-CAP on uPA is of high interest since it is an established prognostic indicator in breast cancer and, together with PAI-1, comprise efficient predictors of distant metastases in a subset of early node-negative breast cancer patients (78, 79). However, these proteases were induced in ER+ cells (mainly uPA) **(** **Figures 10B, C, D** **),** indicating a differential effect of DBD-CAP on the proteolytic potential of breast cancer cells of different ER status. A similar differential effect was also evident for the inflammatory mediators IL-6 and IL-8, which were suppressed in ER- but induced in ER+ breast cancer cells **(** **Figures 10E, F** **)**. Although these findings need further investigation, they clearly demonstrate that CAP has a substantial impact on tumor cell microenvironment, while significant changes in specific matrix effectors seem to occur with regard to the ER expression profile. This seems reasonable given the role of ROS/RNS as major mediators of ECM remodeling during tumor progression (80).

Collectively, our data revealed important DBD-CAP-mediated changes in tumor cell viability and expression of specific matrix effectors not only between breast cancer cells of different ER status (i.e. MCF-7/ER+ *vs* MDA-MB-231/ER- and Hs578T/ER-) but also between cells of the same ER profile (i.e. MDA-MB-231/ER- *vs* Hs578T/ER-), since MDA-MB-231/ER- cells appeared more resistant to the applied DBD-CAP treatments than the Hs578T/ER- cells. This observation implies that different molecular mechanisms and effectors determine tumor cell behavior in the newly-formed plasma-induced microenvironment and this should be considered for the treatment of different breast cancer subtypes. Despite recent advances in breast cancer classification that have resulted in efficient targeted therapies for a large cohort of patients, evidence support that there is a high heterogeneity among breast cancers even between those belonging to the same subtype (81, 82). This is reflected to the differences observed for the two ER- cell lines used in the present study, which could be explained from their different molecular characteristics and origin. The Hs578T originated from a carcinosarcoma of the breast (83) while MDA-MB-231 originated from invasive ductal carcinoma (84). Therefore, a precise classification and a targeted therapy for each individual tumor would highly benefit breast cancer patients.

In conclusion, the present study provides a new perspective to understand the interactions of CAP with the tumor cells and their surrounding microenvironment. Our data suggest that CAP operating under mild conditions inhibits the growth of metastatic breast cancer cells and, at the same time, it regulates the expression of extracellular matrix effectors with established roles in inflammation and cancer. Although these interactions need further investigation, we suggest that CAP could represent an effective therapeutic mean for the treatment of specific breast cancer subtypes with regard to their ER status.

## Data Availability Statement

The original contributions presented in the study are included in the article/**Supplementary Material**, further inquiries can be directed to the corresponding author/s.

## Author Contributions

SS and CA conceived the experiments. A-MC, MT, SM, M-EC, TK, and AC. conducted the experiments. SS and CA analyzed the results. All authors reviewed the manuscript. All authors contributed to the article and approved the submitted version.

## Funding

This research was supported by Grant (project code: 80626) from the Research Committee of the University of Patras, Greece, *via* “C. CARATHEODORI” program. Τhe publication of this article has been financed by the Research Committee of the University of Patras.

## Conflict of Interest

The authors declare that the research was conducted in the absence of any commercial or financial relationships that could be construed as a potential conflict of interest.

## Publisher’s Note

All claims expressed in this article are solely those of the authors and do not necessarily represent those of their affiliated organizations, or those of the publisher, the editors and the reviewers. Any product that may be evaluated in this article, or claim that may be made by its manufacturer, is not guaranteed or endorsed by the publisher.

## Supplementary Material

Supplementary Figure 1 ROS scavenger NAC partly inhibits DBD-CAP-mediated effects on breast cancer cell viability. Morphology of **(A)** MCF-7, **(B)** MDA-MB-231, **(C)** Hs578T breast cancer cells after mild (0.5 kHz/27.6 kV) DBD-CAP (direct, indirect, media change) for 4 min in the absence or presence of NAC (pre-treatment with 5 mM NAC-DMEM for 2 h). Representative phase contrast images captured 24 h after DBD-CAP treatment are shown (magnification, *10X*).
