# Supplementary figures and images for: Cold Atmospheric Plasma Attenuates Breast Cancer Cell Growth Through Regulation of Cell Microenvironment Effectors
Source: Front Oncol. 2022 Jan 17;11:826865. doi: 10.3389/fonc.2021.826865 (PMC8801750; doi:10.3389/fonc.2021.826865)

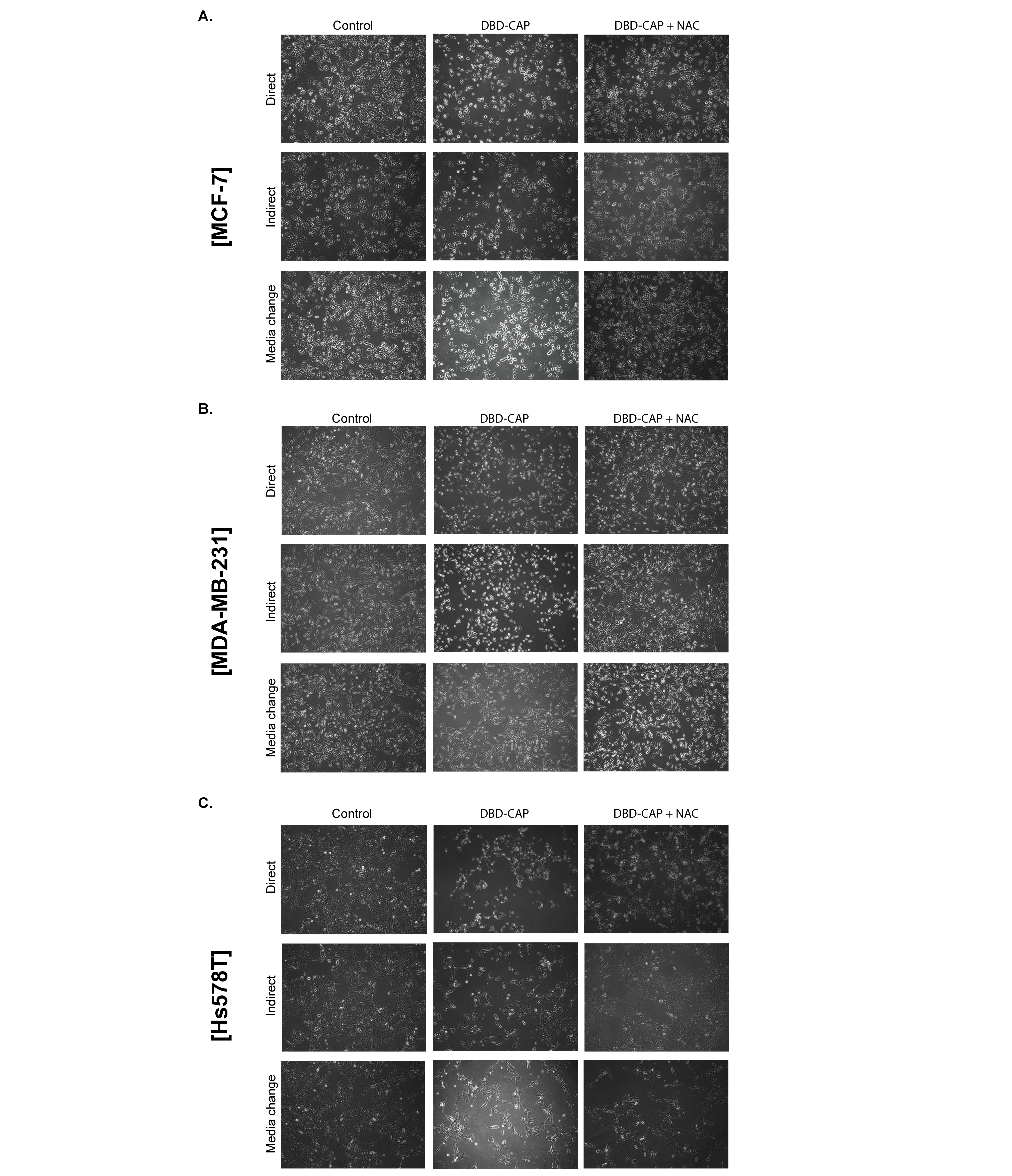

Supplement: Supplementary Figure 1 — ROS scavenger NAC partly inhibits DBD-CAP-mediated effects on breast cancer cell viability. Morphology of (A) MCF-7, (B) MDA-MB-231, (C) Hs578T breast cancer cells after mild (0.5 kHz/27.6 kV) DBD-CAP (direct, indirect, media change) for 4 min in the absence or presence of NAC (pre-treatment with 5 mM NAC-DMEM for 2 h). Representative phase contrast images captured 24 h after DBD-CAP treatment are shown (magnification, 10X). [file Image_1.tif]
